# Identifying cross-category relations in gene ontology and constructing genome-specific term association networks

**DOI:** 10.1186/1471-2105-14-S2-S15

**Published:** 2013-01-21

**Authors:** Jiajie Peng, Jin Chen, Yadong Wang

**Affiliations:** 1 School of Computer Science and Technology, Harbin Institute of Technology, Harbin, China; 2 MSU-DOE Plant Research Laboratory, Michigan State University, East Lansing, MI 48824, USA; 3 Department of Computer Science and Engineering, Michigan State University, East Lansing, MI 48824, USA

## Abstract

**Background:**

Gene Ontology (GO) has been widely used in biological databases, annotation projects, and computational analyses. Although the three GO categories are structured as independent ontologies, the biological relationships across the categories are not negligible for biological reasoning and knowledge integration. However, the existing cross-category ontology term similarity measures are either developed by utilizing the GO data only or based on manually curated term name similarities, ignoring the fact that GO is evolving quickly and the gene annotations are far from complete.

**Results:**

In this paper we introduce a new cross-category similarity measurement called CroGO by incorporating genome-specific gene co-function network data. The performance study showed that our measurement outperforms the existing algorithms. We also generated genome-specific term association networks for yeast and human. An enrichment based test showed our networks are better than those generated by the other measures.

**Conclusions:**

The genome-specific term association networks constructed using CroGO provided a platform to enable a more consistent use of GO. In the networks, the frequently occurred MF-centered hub indicates that a molecular function may be shared by different genes in multiple biological processes, or a set of genes with the same functions may participate in distinct biological processes. And common subgraphs in multiple organisms also revealed conserved GO term relationships. Software and data are available online at http://www.msu.edu/˜jinchen/CroGO.

## Background

Gene Ontology (GO) is one of the most popular languages for describing and categorizing attributes of biological entities, and utilizes three key categories that are shared by all organisms [1]: molecular function (MF; biochemical function of the gene product), biological process (BP; the biological process to which the gene product contributes) and cellular component (CC; location of the gene product in the cell). To automatically discover novel biological relationships between GO terms, the measurement of term similarities has been extensively studied [2-5], and it remains an active research area in semantic comparison and search [6]. However, most of these similarity methods cannot measure semantic similarities between terms in the different root ontology categories. Although the three root GO categories (MF, BP and CC) are structured as independent ontologies, their biological relationships (especially between BP and MF terms) may provide useful evidence for gene annotation [7]. More importantly, discovering such cross-category associative relationships may help researchers conduct biological reasoning and generate biological hypotheses. For example, if a set of gene products that have the same molecular function often participate in multiple biological processes, then these biological processes may be tightly associated with each other at metabolic level via this molecular function.

**Figure 1:**
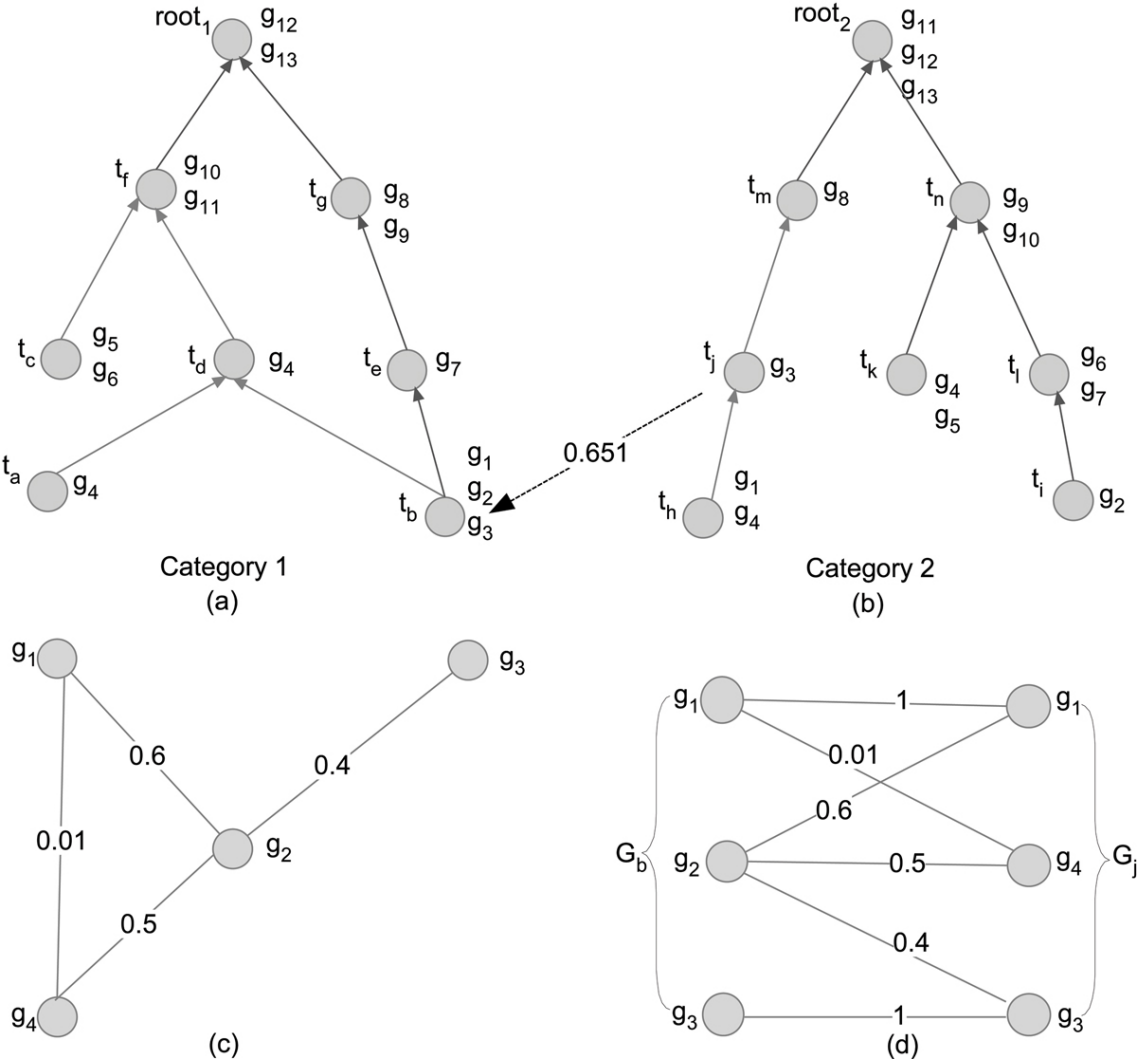
**An example of two GO categories, gene co-function network and gene set association**. (a and b) An example of two GO categories, in which each node is a GO term, each edge represents a conceptual relation between two terms, and {*g*_1_...*g*_13_} is the set of genes annotated to corresponding terms. (c) An example of gene co-function network, in which each node is a gene, each edge represents the functional associations between the genes, and the confidence score at each edge measures the probability of an interaction to represent a true functional linkage between two genes. (d) The gene set association between gene set *G_b _*and *G_j_*.

To the best of our knowledge, the state-of-art algorithms to identify strong association relationships across GO categories can be classified into two categories: Association Rule Mining (ASR) and Text Mining such as Vector Space Model (VSM). Several algorithms have been developed to identify strong association relationships across GO categories [6-8]; For example, the similarity between terms *t_b _*and *t_j _*that belong to two different GO categories are shown in Figure 1(a, b). A classic data mining algorithm called association rule mining (ASR) was adopted by Bodenreider *et al *[8] and Kumar *et al *[6] to compute cross-category GO term similarity *Sim_ASR_*(*t*_1_, *t*_2_), where terms *t*_1 _and *t*_2 _are in category *C*_1 _and *C*_2 _respectively. Based on these approaches, a ready-for-use inter-category GO structure has been constructed by Myhre *et al *[7] and is provided as an addition to GO. Note that the ASR-based term associations are directional, *i.e.*, *Sim_ASR _*(*t*_1_, *t*_2_) may be different to *Sim_ASR _*(*t*_2_, *t*_1_). However, the "shallow annotation" problem [9] was ignored in the ASR-based measures, because if both *t*_1 _and *t*_2 _are very close to the root of *C*_1 _and *C*_2_, chances are high that both *Sim_ASR _*(*t*_1_, *t*_2_) and *Sim_ASR _*(*t*_2_, *t*_1_) are high regardless of whether they are biologically related, since the terms near the root contain almost all of the genes after propagation [5]. As a result, term pairs that are at very shallow levels of the GO hierarchy (*e.g.*, "response to stimulus") can yield very high semantic similarities, and such pairs are not distinguishable from high-scoring pairs that are "deep" at GO hierarchical structure [10].

To avoid the "shallow annotation" problem, Bodenreidar *et al *proposed a Vector Space Model (VSM)based measure [8], which considers the semantic weight of each gene product. VSM has been widely used in information retrieval applications for calculating the similarities among documents that can be described with vectors [11]. Mathematically, given two vectors *v*_1 _and *v*_2 _consisting of binary values indicating the presence (1) and absence (0) of an association between a term (*t*_1 _or *t*_2_) and a gene, the term similarity *Sim_VSM _*(*t*_1_, *t*_2_) can be calculated with a weight-adjusted cosine similarity equation, representing the angle between *v*_1 _and *v*_2_. However, using this approach, the resulting cross-category association relationships are undirected because of *Sim_VSM _*(*t*_1_, *t*_2_) ≡ *Sim_VSM _*(*t*_2_, *t*_1_). Biologically, if a MF term points to a BP term, the MF term *is involved in *the BP term [7]; and if a BP term points to a MF term, the BP term *is realized by *the MF term [6]. Therefore, different directions of the term relationships indicate different biological meanings. Since the VSM-based measure treats *v*_1 _and *v*_2 _equally, the order of the resulting biological associations are lost. Furthermore, the VSM-based measure heavily relies on the overlapped genes of two target terms, ignoring the fact that the annotations are far from complete, *e.g.*, only 28.1% of human genes have at least one non-IEA annotation [12], leading to inaccurate term similarity scores.

There have been many studies which have shown that integrating several different broad types of data can significantly improve the results of bioinformatics methods [13,14], but no such method exists for GO analyses. Starting with the intuition that the incorporation of extra biological information may improve the performance of a cross-category term similarity measure, we propose a new algorithm, Cross-Category Gene Ontology Measurement (CroGO), for calculating the similarity between two cross-category terms by effectively incorporating genome-specific gene co-function network data. Compared to the existing algorithms, CroGO has the following advantages:

1. CroGO incorporates the information from gene co-function networks, which are widely believed to be good complements to GO for understanding the associations between biological concepts. The co-function networks have been constructed using extensive gene expression and protein interaction data containing millions of individual observations from DNA microarrays, physical protein interactions, genetic interactions, literature, and comparative genomics methods [15-17]. Therefore, numerous new cross-ontology associations can be learned by incorporating the co-function networks into a model.

2. Our algorithm determins the directions of term relationships by considering the GO hierarchical structure, while the existing methods either ignores the directions or simply defines the directions by using the different numbers of genes annotated to two terms.

3. The "shallow annotation" problem has been avoided in CroGO by considering the specificity of GO terms, while the ASR-based measures and some statistical measures, *e.g.*, χ^2^-test, may mix shallow-level term pairs with term pairs that are deep at GO hierarchical structure.

4. The term association network generated with CroGO is genome-specific, from which conserved term associations may suggest vital functional connections, and unique term associations in certain organisms may suggest genome-specific functions even for homolog genes. While the term associations generated with lexical approaches [7,8,18] that test whether one term is a substring in the other term are universal for all kinds of organisms.

## Method

To measure the similarity between the terms in different GO categories, CroGO has three steps. First, the association between two sets of genes that are annotated to any two given GO terms is calculated. Second, the gene annotations and gene set associations are integrated to calculate the pair-wise term similarity. Third, the directions of all the pair-wise term relationships are inferred with a GO structure based approach.

### Step 1: Gene set association

To measure the association between two gene sets *G*_1 _and *G*_2 _that are annotated to terms *t*_1 _and *t*_2 _in GO categories *C*_1 _and *C*_2 _respectively, we define Gene Set Association (GSA) by taking into consideration the weighted edges in a gene co-function network *N*. Nodes in *N *represent genes and edges represent functional interactions between genes, and every edge is associated with a confidence score that measures the probability of interaction. An illustrative example of a gene co-function network with four genes is shown in Figure 1(c). GSA is defined as:

(1) $$GSA\left(G_{1},G_{2}\right)=\frac{\left|G_{1}\cup G_{2}|-|G_{1}-G_{2}|-|G_{2}-G_{1}\right|}{\left|G_{1}\cup G_{2}\right|}$$

where *G*_1 _(or *G*_2_) is the set of genes annotated to *t*_1 _(or *t*_2_), |*X| *represents the size of set *X*, *G*_1 _∪ *G*_2 _is the union of *G*_1 _and *G*_2_, and |*G*_1 _− *G*_2_| is defined as:

(2) $$\left|G_{1}-G_{2}\right|=\left|G_{1}\right|-\underset{g_{i}\in G_{1}}{\sum }\left(1-\underset{g_{j}\in G_{2}}{\prod }\left(1-w_{ij}\right)\right)$$

where *w_ij _*is functional similarity score between genes *g_i _*and *g_j _*in gene co-function network *N*:

(3) $$w_{ij}=\left\{\begin{array}{cc} 1, & i=j\\ 0, & <g_{i},g_{j}>\notin N\\ cof\left(g_{i},g_{j}\right) & else \end{array}\right)$$

where <*g_i_*, *g_j _*> is an edge in *N*, and *cof *(*g_i_*, *g_j_*) is the likelihood of the functional interaction between *g_i _*and *g_j _*in *N*, and *cof *(*g_i_*, *g_j_*) ∈ [0, 1].

In Equation 2, the right part represents the semantic overlapping between *G*_1 _and *G*_2_. If gene set *G*_1 _and *G*_2 _are the same, then |*G*_1 _− *G*_2_| = 0, consequently *GSA*(*G*_1_, *G*_2_) = 1; and if *G*_1 _and *G*_2 _do not have any overlap and there is no linkage between the gene sets in *N*, then |*G*_1 _− *G*_2_| = |*G*_1_|, consequently *GSA*(*G*_1_,*G*_2_) = 0. In summary, the gene set association score *GSA*(*G*_1_, *G*_2_) represents the association between two gene sets *G*_1 _and *G*_2 _based on the shared genes and the gene associations in a co-function network.

### Step 2: Pair-wise similarity measure

Given two GO terms *t*_1 _and *t*_2 _from different GO categories *C*_1 _and *C*_2_, the term similarity *Sim*(*t*_1_, *t*_2_) is defined with the integration of GO structure, gene annotations and co-function network:

(4) $$Sim\left(t_{1},t_{2}\right)=GSA\left(G_{1},G_{2}\right)\cdot \sqrt{\left(1-\frac{\left|G_{1}\right|}{\left|G_{C_{1}}\right|}\right)\cdot \left(1-\frac{\left|G_{2}\right|}{\left|G_{C_{2}}\right|}\right)}$$

where *GSA*(*G*_1_, *G*_2_) is calculated with Equation 1, and $G_{C_{1}}$ and $G_{C_{2}}$ are the sets of all the genes involved in category *C*_1 _and *C*_2 _respectively.

In Equation 4, the first part, *GSA*(*G*_1_, *G*_2_), represents the association between the gene sets annotated to the terms *t*_1 _and *t*_2_, which takes advantage of both the prior knowledge deposited in GO and the experimental gene-gene associations summarized in the gene co-function networks. The second part describes the specificity of both terms by considering the level of generality of *t*_1 _and *t*_2 _in their own GO categories to avoid the "shallow annotation" problem.

### Step 3: Term pair direction assignment

We look for the directions of the relationships between two terms with a pruning approach. First, all-by-all term similarities are calculated with Equation 4 and term pairs with high similarity scores are saved with bi-directions. Then for each term *t *and a set of terms *T *that connect to *t*, we remove the edge direction from *t *to term *t' *only if there exists another term *t'' *such that *t *is an ancestor of *t'' *(*t', t'' *∈ *T *). In the end, if both directions at edge <*t*, *t' *> are removed, then edge <*t*, *t' *> shall be removed as well.

In the example in Figure 1(a, b), *t_b _*and *t_j _*are two terms in category *C*_1 _and *C*_2_, and the genes annotated to them are shown in Figure 1(d), in which the functional similarity scores of any two genes are calculated with a co-function network in Figure 1(c). To calculate the directional association between *t_b _*and *t_j_*, we first calculate all-by-all similarity scores, e.g., *Sim*(*t_b_*, *t_j_*) = 0.651, *Sim*(*t_b_*, *t_h_*) = 0.465, etc., using Equation 4, and then apply a user defined threshold (say, 0.45) to filter term relationships with low scores. In the third step, the direction from *t_b _*to *t_j _*is removed, since *t_j _*is an ancestor of *t_h_*, and the direction from *t_j _*to *t_b _*is retained, because there is no child of *t_b _*that also connects to *t_j _*and *Sim*(*t_b_*, *t_h_*) is greater than the threshold. Finally, we conclude that *t_j _*points to *t_b _*with similarity score 0.651.

## Results

In the test experiments, we focused on identifying the relationships between the BP and MF terms. To show the significance of CroGO, we first compared its results with both the ASR-based and VSM-based measures on a small gold-standard set generated with known reaction-to-pathway relationships on yeast. Then we constructed the MF-BP cross-ontology term association network of yeast, and evaluated it with evidences from the manually-curated yeast pathway database. Finally, we studied the conservation of cross-ontology associations by comparing the yeast and human term association networks.

The GO data and gene annotations was downloaded from GO website in February, 2012, in which only the annotations with non-IEA evidences were used [1]. The gene co-function network was obtained from YeastNet [15], which has 102,803 linkages among 5,483 genes. The co-function score of each linkage was normalized between 0 and 1. CroGO was developed with Java JDK 1.6 and JUNG library [19].

### Performance comparison on gold-standard set

To compare the performance of CroGO with the existing measures with confirmed biological knowledge, we first generated a small "gold-standard" set based on the known reaction-to-pathway relationships [7] in yeast in three steps: 1) we associate a BP term to a metabolic pathway if the pathway corresponds directly to a GO-defined biological process; 2) a metabolic pathway is associated to several Enzyme Commission (EC) numbers if the enzymes catalyze the pathway; and 3) we link a EC number to a MF term with the official GO translations [20,21]. In the end, a small gold-standard set of reliable MF-BP associations was obtained via the known metabolic pathways and EC numbers. In YeastCyc [22], 71 out of the total 187 pathways match exactly to BP terms and have at least one EC number associated to them. From these pathways, 175 MF-BP pairs were identified and saved as the yeast gold-standard set (Additional file 1). We also randomly selected term pairs to construct a random set, which is 10 times larger than the gold-standard set.

**Figure 2:**
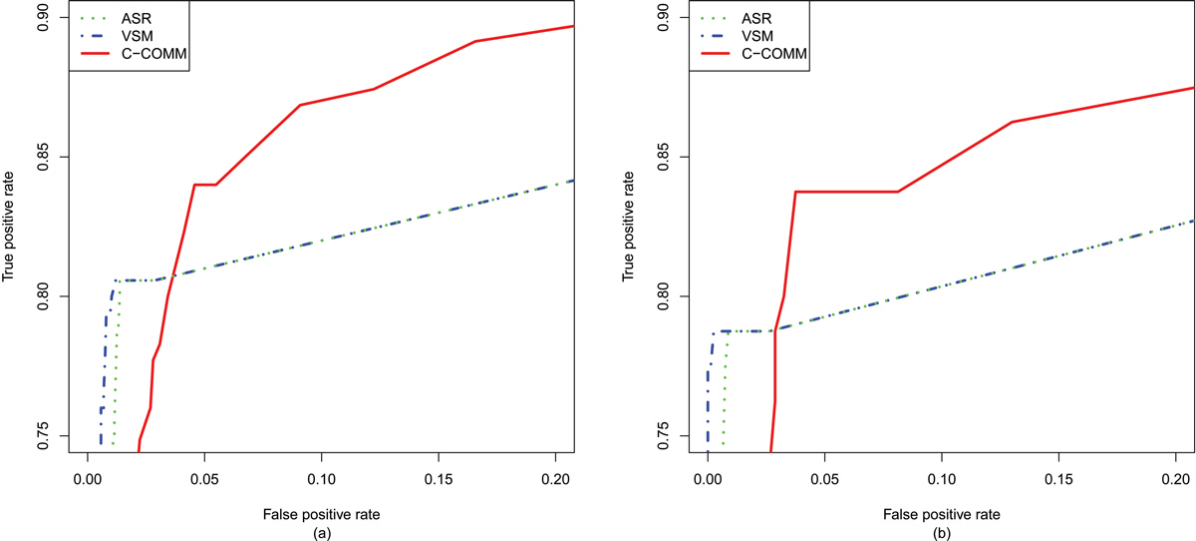
**ROC curves for the experimental results on the gold-standard sets**. ROC curves for the experimental results on the gold-standard sets of yeast (a) and human (b) calculated with CroGO (red), VSM (blue) and ASR (green). ASR and VSM based measures have very similar trends, and are overlapping for most of the visible portion of the ROC curves.

We calculated pair-wise term similarities for the term pairs in the gold-standard set and the term pairs in the random set using CroGO, and compared its performance with the ASR and VSM based measures by drawing a receiver operating characteristic (ROC) curve [23] for each measure. The ROC curves in Figure 2(a) showed clearly that CroGO has the best performance. As shown in Table 1, when the false positive threshold was set to 15%, the true positive rate of CroGO is 88%, while the true positive rates of the ASR and VSM based measures are both 83%. This analysis also showed that 102 more MF-BP pairs were recognized by CroGO than the ASR and VSM based measures when the number of true positives equals the number of false positives. This indicates that by incorporating the co-function network, CroGO has produced better coverage than the other measures by recognizing more gene associations between genes which are annotated to the gold-standard connected GO terms. In addition, the same experiments were applied on human data, and the results is consistent to the yeast data (see details in the conserved association section).

**Table 1:** The performance study on yeast and human gold-standard sets.

Organism	Measure	No. of term pairs (when FP = TP)	TP rate (when FP = 5%)	TP rate (when FP = 10%)	TP rate (when FP = 15%)
Yeast	ASR	50	81%	82%	83%
	VSM	50	81%	82%	83%
	CroGO	**152**	**84%**	**87%**	**88%**

Human	ASR	21	79%	80%	81%
	VSM	21	79%	80%	81%
	CroGO	**67**	**84%**	**85%**	**87%**

The performance study of CroGO, VSM and ASR based measures on yeast and human gold-standard sets.

### Genome-specific MF-BP association network

To demonstrate the practical use of CroGO and provide researchers a platform to enable a more consistent use of GO, we linked biological concepts by generating a genome-specific term association network. Mathematically, we constructed bipartite graph Ω (*M*, *B*, *E*) where *M *and *B *are sets of nodes representing MF and BP terms respectively; edge *e *<*m*, *b *> in *E *indicates that *m *is involved in *b*; and edge *e *<*b*, *m *> indicates *b *is realized by *m *(*b *∈ *B *and *m *∈ *M*). We constructed the highly reliable MF-BP association network Ω*_yeast _*(*M*, *B*, *E*) by comparing all the MF and BP terms with CroGO and adopting a strict z-score cutoff (in this paper we required *z *− *score *> 8.0). Therefore, all the edges in the MF-BP association network are statistically significant. In the end, network Ω*_yeast _*(*M*, *B*, *E*) has 613 MF terms, 843 BP terms and 1,485 edges. As shown in Additional file 2 the yeast association network Ω*_yeast _*(*M*, *B*, *E*) consists of many small disconnected graphs.

We evaluated the whole network performance of Ω*_yeast _*(*M*, *B*, *E*) with an GO enrichment approach. A biological process usually contains multiple biochemical reactions. The genes in two adjacent reactions should have similar BP annotations, because the product of one reaction is the substrate of the other one. With this criterion, we evaluate the performance of CroGO by testing whether a given MF term that is linked to a reaction *r *connects to all the BP terms that are enriched in the adjacent reactions of *r*. By extracting reaction information from YeastCyc, we found 82 valid MF terms in Ω*_yeast _*(*M*, *B*, *E*), and among them 56 MF terms (67.1%) were connected to enriched BP terms. For performance comparison, we constructed two extra yeast MF-BP association networks using the ASR and VSM based approaches with the same z-score cutoff. This test showed that in the VSM-based result, only 25 out of the valid 43 MF terms (58.1%) were connected to enriched BP terms; and due to the "shallow annotation" problem, there is no valid MF term in the ASR-based result. Again, these results indicate that CroGO is superior to the existing measures in constructing term association networks.

**Figure 3:**
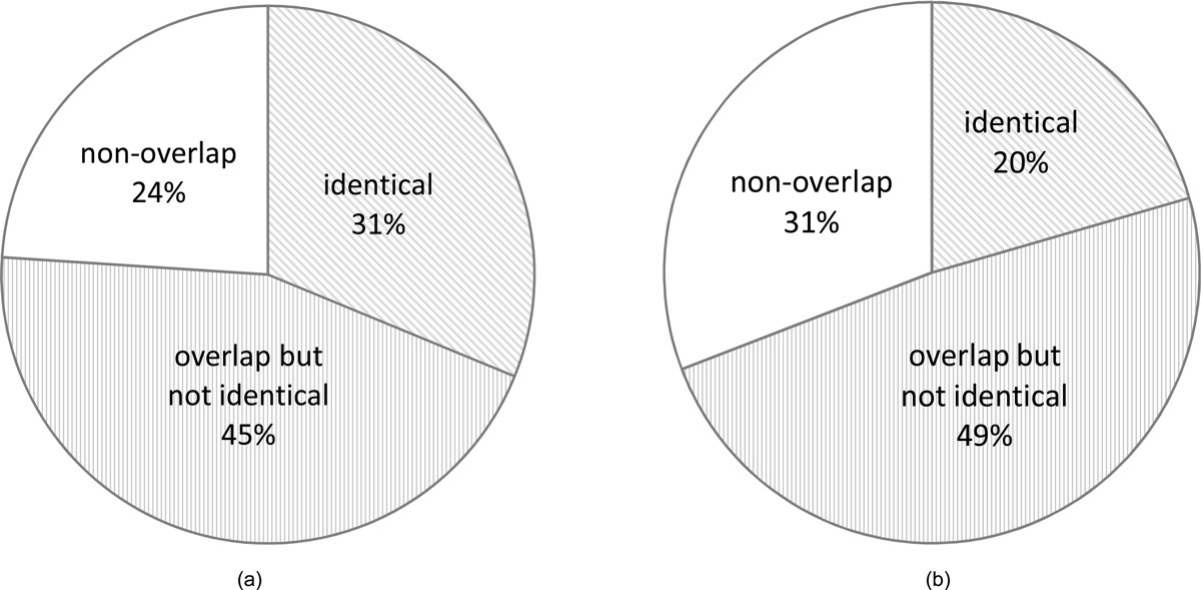
**The edges distribution in the genome-specific term association network of yeast (a) or human (b)**. The edges distribution in the genome-specific term association network of yeast (a) or human (b). The three categories are "identical", "non-overlap" and "overlap but not identical". It indicates a significant part ("non-overlap") of the networks can only be identified by CroGO because of the incorporation of extra biological information from the co-function networks.

An edge <*t*_1_, *t*_2 _> in the term association network can be classified into one of the three categories: "identical" (*G*_1 _= *G*_2_), "non-overlap" (*G*_1 _∩ *G*_2 _= ∅) and "overlap but not identical" (*G*_1 _∩ *G*_2 _≠ ∅ and *G*_1 _≠ *G*_2_), where *G*_1 _(or *G*_2_) is the set of genes annotated to term *t*_1 _(or *t*_2_). Figure 3(a) shows that 356 term relationships (24%) in the "non-overlap" category can only be found by CroGO because of the incorporation of extra biological information from a co-function network. The top 20 term associations in the "overlap but not identical" category and the top 20 term associations in "non-overlap" category are listed in Table 2 and 3. In these term associations, 24 were supported by the existing biological studies or lexical matching on term definition, and the rest 16 are new conceptual connections that cannot be found in any literature. For example, MF term "endopeptidase activator activity" is assigned to PRE1 and PUP3 that hydrolyze nonterminal peptide bonds in polypeptides; and BP term "proteasome core complex assembly" means the aggregation, arrangement and bonding together of a mature, active 20S proteasome core particle complex that does not contain any regulatory particles, and it is annotated to PRE2 and PRE9. Clearly these two terms do not have any common genes. However, their annotated genes are tightly connected with each other in the co-function network because the gene products were found in the same protein complexes from affinity purification/mass spectrometry data [15], and an endogenous activator of the protease was also identified [24]. Therefore, the two remote concepts are connected by CroGO, suggesting that endopeptidase activators are involved in proteasome core complex assembly.

**Table 2:** Top 20 term associations in category "overlap but not identical" that were identified by CroGO.


**MF Name**	**BP Name**	**Evidence**

polynucleotide adenylyltransferase activity	ncRNA polyadenylation	NEW
TFIIF-class binding TF activity	regulation of transcription-coupled nucleotide-excision repair	REF [37]
TFIIF-class binding TF activity	positive regulation of transcription elongation from Pol I promoter	REF [38]
TFIIF-class binding TF activity	regulation of transcription elongation from Pol I promoter	REF [38]
TFIIF-class binding TF activity	positive regulation of histone H3-K36 trimethylation	NEW
TFIIF-class binding TF activity	regulation of histone H3-K36 trimethylation	NEW
TFIIF-class binding TF activity	positive regulation of histone H3-K36 methylation	NEW
TFIIF-class binding TF activity	regulation of nucleotide-excision repair	REF [39]
TFIIF-class binding TF activity	regulation of histone H2B ubiquitination	REF [40]
TFIIF-class binding TF activity	positive regulation of phosphorylation of Pol II C-terminal domain serine 2 residues	NEW
TFIIF-class binding TF activity	regulation of phosphorylation of Pol II C-terminal domain serine 2 residues	NEW
TFIIF-class binding TF activity	regulation of histone H2B conserved C-terminal lysine ubiquitination	NEW
IMP dehydrogenase activity	GTP biosynthetic process	REF [41]
hydrogen ion transporting ATP synthase activity, rotational mechanism	ATP biosynthetic process	LEXICAL
RNA-directed RNA polymerase activity	tRNA transcription from Pol III promoter	LEXICAL
RNA-directed RNA polymerase activity	tRNA transcription	NEW
protein prenyltransferase activity	protein geranylgeranylation	REF [42]
second spliceosomal transesterification activity	generation of catalytic spliceosome for second transesterification step	NEW
oxoglutarate dehydrogenase activity	2-oxoglutarate metabolic process	LEXICAL
peptide alpha-N-acetyltransferase activity	N-terminal protein amino acid acetylation	REF [43]

Top 20 term associations in category "overlap but not identical" that were identified by CroGO. In the list, 8 term associations are supported by the existing biological studies, 3 are supported by the lexical matching on term definition, and the rest 7 are new conceptual connections that cannot be found in any literature. Only 3 of the term associations can be identified by the VSM or ASR based measures.

**Table 3:** Top 20 term associations in category "non-overlap" that were identified by CroGO.

MF Name	BP Name	Evidence
endopeptidase activator activity	proteasome core complex assembly	NEW
TFIIF-class binding TF activity	regulation of histone H3 K79 methylation	NEW
RNA-directed RNA polymerase activity	DNA-dependent transcriptional start site selection	LEXICAL
RNA-directed RNA polymerase activity	transcriptional start site selection at Pol II promoter	LEXICAL
single base insertion or deletion binding	chiasma assembly	REF [26]
double-strand/single-strand DNA junction binding	chiasma assembly	REF [26]
double-stranded telomeric DNA binding	gene conversion at mating-type locus, DNA double-strand break processing	NEW
G-quadruplex DNA binding	gene conversion at mating-type locus, DNA double-strand break processing	NEW
very long-chain fatty acid-CoA ligase activity	long-chain fatty-acyl-CoA metabolic process	REF [27]
very long-chain fatty acid-CoA ligase activity	fatty-acyl-CoA metabolic process	REF [27]
very long-chain fatty acid-CoA ligase activity	acyl-CoA metabolic process	REF [27]
single base insertion or deletion binding	meiotic heteroduplex formation	NEW
guanine/thymine mispair binding	chiasma assembly	NEW
TFIIE-class TF binding	negative regulation of ribosomal protein gene transcription from Pol II promoter in response to chemical stimulus	REF [28]
TFIIE-class binding TF activity	negative regulation of ribosomal protein gene transcription from Pol II promoter in response to chemical stimulus	REF [28]
Hsp90 protein binding	positive regulation of telomere maintenance via telomerase	NEW
Hsp90 protein binding	positive regulation of telomere maintenance	REF [29]
Hsp90 protein binding	positive regulation of homeostatic process	REF [30]
aldehyde dehydrogenase activity	beta-alanine metabolic process	REF [31]
aldehyde dehydrogenase activity	beta-alanine biosynthetic process	REF [31]

Top 20 term associations in category "non-overlap" that were identified by CroGO. In the list, 11 term associations are supported by the existing biological studies, 2 are supported by the lexical matching on term definition, and the rest 7 are new conceptual connections that cannot be found in any literature. None of the term associations can be identified by the VSM or ASR based measures.

**Figure 4:**
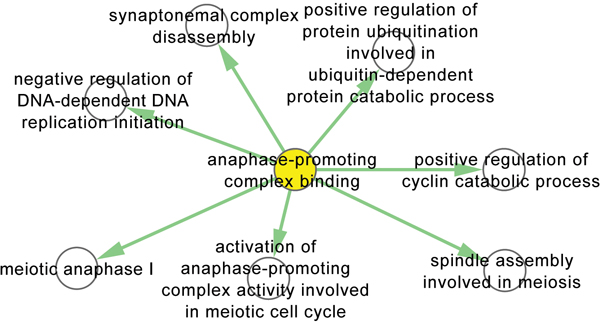
**A case study of a MF-centered topological pattern found in the yeast MF-BP association network**. A case study of a MF-centered topological pattern found in the yeast MF-BP association network. The yellow and white nodes are MF and BP term respectively.

An interesting topological pattern found in the yeast MF-BP association network is the frequently occurred MF-centered hub. As the case study shown in Figure 4, the function "anaphase-promoting complex binding" is involved in seven different biological processes in yeast from metaphase to anaphase during mitosis, including "activation of anaphase-promoting complex activity involved in meiotic cell cycle", suggesting that anaphase-promoting complex is key for mitotic cyclins and anaphase inhibitory protein degradation, thereby triggering sister chromatid separation and exit from mitosis [25]. In summary, by connecting remote concepts, researchers are able to conduct advanced biological reasoning and generate interesting biological hypotheses.

### Conserved MF-BP associations

To explore which part of the MF-BP association network is conservative and which part is varying from one organism to another, we constructed the human MF-BP association network Ω*_human _*(*M*, *B*, *E*) by comparing all the MFs and BPs that are annotated to at least one human gene and adopting the same z-score cutoff (8.0). Ω*_human _*(*M*, *B*, *E*) has 1,209 MF terms, 2,250 BP terms and 5,138 edges, among which 1,583 edges are between terms that have no overlap on their annotated genes (see Figure 3(b)), indicating that at least 30% of the edges are solely contributed by the human co-function network HumanNet [17] (which has 476,399 linkages among 16,243 genes). Unlike the yeast MF-BP association network, the human MF-BP association network Ω*_human _*(*M*, *B*, *E*) showed in Additional file 3 has a large subgraph occupying ~ 50% of the total edges.

To evaluate the performance of CroGO on human data, we generated the human gold-standard set consisting of 80 MF-BP pairs (Additional file 4) from the human pathway data humanCyc [32] and a random set which was 10 times larger. The ROC curves in Figure 2(b) showed that CroGO has the best performance. When the false positive threshold was set to 15%, the true positive rate of CroGO is 87%, while the true positive rates of the ASR and VSM based measures are both 81%. This analysis also showed that 46 more MF-BP pairs were able to be recognized by CroGO than the ASR and VSM based measures when the number of true positives equals the number of false positives, indicating CroGO has outperforms the other measures on human data as well as on the yeast data.

**Figure 5:**
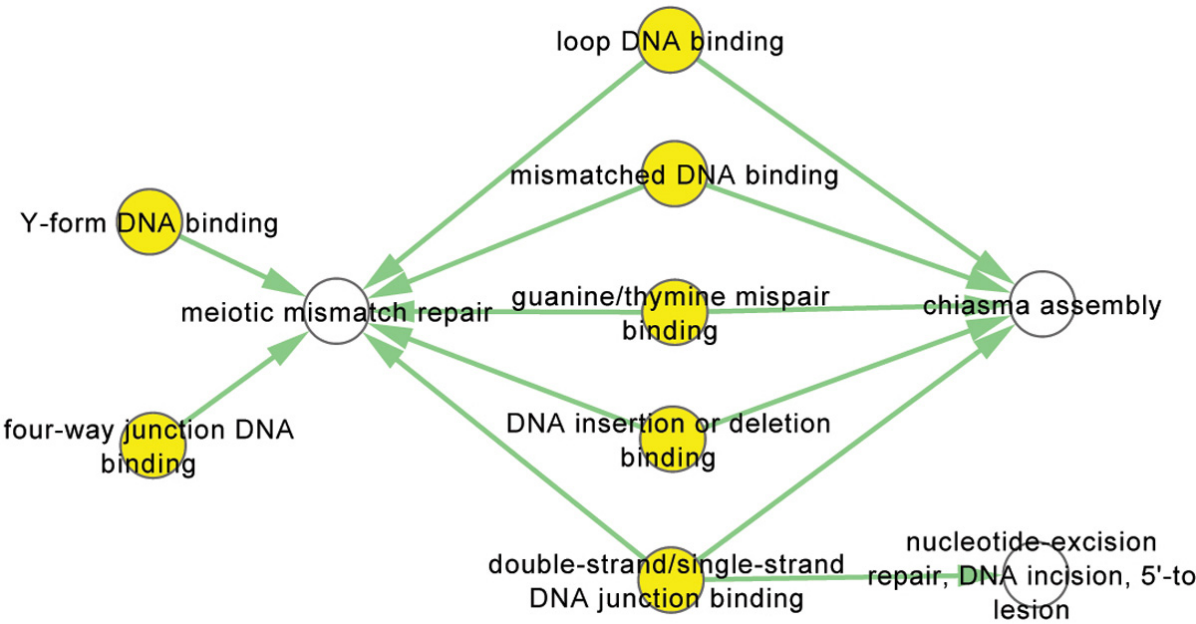
**An example that five different types of DNA binding proteins are involved in biological processes meiotic mismatch repair and chiasma assembly in both human and yeast**. An example that five different types of DNA binding proteins are involved in biological processes meiotic mismatch repair and chiasma assembly in both human and yeast. The yellow and white nodes are MF and BP term respectively.

Additional file 2 and Additional file 3 show that the MF-BP relations in human are much more complex than in yeast, yet their common MF-BP relationships still reveal interesting conserved patterns in the long evolution process. Figure 5 shows an example in which five different types of DNA binding proteins are involved in the biological processes "meiotic mismatch repair" and "chiasma assembly" in both human and yeast. As an evidence, the genes and concepts that connect "DNA binding", "meiotic mismatch repair" and "chiasma assembly" are drawn with BioGraph [33] in Additional file 5. Mismatch repair proteins are a highly diverse group of proteins that interact with numerous DNA structures during DNA repair and replication [34]. In this protein group, three MSH proteins form an active protein complex to play an essential role in DNA repair by fixing mistakes that are made when DNA is copied in preparation for cell division [35]. MSH6, a DNA mismatch repair homolog of human MutS protein in yeast, plays a role in binding double-stranded DNA and in four-way junction DNA Binding [36]. Probably because of the importance of these DNA-binding proteins, their functions and their roles in the two biological processes are conserved during evolution.

## Conclusions

In each GO category, the ontology is structured as a directed acyclic graph to reflect the complex hierarchy of biological events and locations, but the thee root GO categories are structured as independent ontologies. By connecting MF term to BP terms, the low-granularity MFs can be related to higher-granularity BPs, providing useful evidence for improved biological reasoning.

The similarity measure between GO terms in different categories has been the focus of other studies. However, existing measurements are either developed by utilizing only the GO data, or have been based on the similarity between term names.

Starting with the intuition that the incorporation of extra biological information may improve the performance of a cross-category term similarity measure, we propose a new algorithm called CroGO for calculating the similarity between two cross-category terms by incorporating gene co-function network data. Compared to the existing algorithms, CroGO can clearly identify more biologically verified cross-category term relationships, since it utilizes extra biological information that is good complement to GO for understanding the associations between biological concepts. And the ROC curves on small gold-standard sets of human and yeast indicate CroGO can identify term associations more precisely.

To demonstrate the practical use of CroGO and provide researchers a platform to enable a more consistent use of GO, the genome-specific term association networks of yeast and human were generated. In these networks, we found that the frequently occurred MF-centered hub is an interesting topological pattern as it may indicate a molecular function could be shared by different genes in multiple biological processes, or a set of genes with the same functions may be a common component belonging to distinct biological processes. From the topological view, the human association network is much more complex than the yeast term association network. And their common MF-BP relationships reveal evolutionary conserved patterns, indicating important functional associations.

Note that CroGO uses a gene co-function network as part of its input. Therefore, in the condition that such gene co-function does not exist, CroGO is not applicable. In the future, we will extend CroGO to automatically generate the co-function network from existing of gene expression or protein-protein interaction data that a user specified. Second of all, different directions of the term relationships indicate different biological meanings. A relationship from a MF term to a BP term means the MF term is involved in the BP term; and the reverse direction indicates the BP term is realized by the MF term. We will study the directions between BP and MF terms in the future work. And we also would like to extend CroGO to study the relationships between all the three GO categories and apply it on other biological/medical ontologies. Furthermore, we will develop more advanced network biology approaches, such that genome, proteome, metabolome and other -omics data can be jointly analyzed to understand cross-ontology relationships.

## Competing interests

The authors declare that they have no competing interests.

## Authors' contributions

**JC **conceived the project. **JP**, **JC **and **YW **designed the algorithm and experiments. **JP **implemented the algorithm and finished the experiments.

## Declarations

The publication costs for this article were funded by the corresponding author's institution.

This article has been published as part of *BMC Bioinformatics *Volume 14 Supplement 2, 2013: Selected articles from the Eleventh Asia Pacific Bioinformatics Conference (APBC 2013): Bioinformatics. The full contents of the supplement are available online at http://www.biomedcentral.com/bmcbioinformatics/supplements/14/S2.

## Supplementary Material

Additional file 1 **Gold-standard set on Yeast**. 175 gold-standard set of MF-BP relationships in yeast. Click here for file

Additional file 2 **MF-BP Association Network of yeast**. MF-BP Association Network of yeast. The nodes represent terms and the edges represent the term associations discovered by CroGO. The yeast MF-BP association network consists many small disconnected graphs. Click here for file

Additional file 3 **MF-BP Association Network of human**. MF-BP Association Network of human. The nodes represent terms and the edges represent the associations discovered by CroGO. The human MF-BP association network has a large subgraph occupying 50% of total edges. Click here for file

Additional file 4 **Gold-standard set on Human**. 80 gold-standard set of MF-BP relationships in human. Click here for file

Additional file 5 **genes and concepts that connect "DNA binding", "meiotic mismatch repair" and "chiasma assembly"**. The genes and concepts that connect "DNA binding", "meiotic mismatch repair" and "chiasma assembly". The figure was generated with BioGraph [40]. Click here for file
