# Supplementary figures and images for: Identifying cross-category relations in gene ontology and constructing genome-specific term association networks
Source: BMC Bioinformatics. 2013 Jan 21;14(Suppl 2):S15. doi: 10.1186/1471-2105-14-S2-S15 (PMC3549802; doi:10.1186/1471-2105-14-S2-S15)

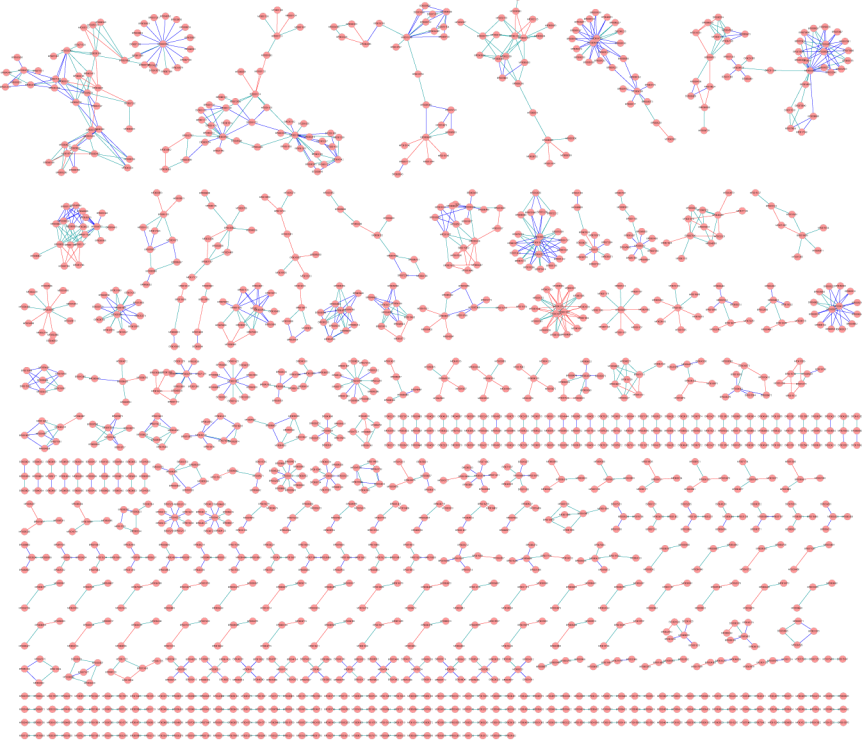

Supplement: Additional file 2 — MF-BP Association Network of yeast. MF-BP Association Network of yeast. The nodes represent terms and the edges represent the term associations discovered by CroGO. The yeast MF-BP association network consists many small disconnected graphs. [file 1471-2105-14-S2-S15-S2.pdf]

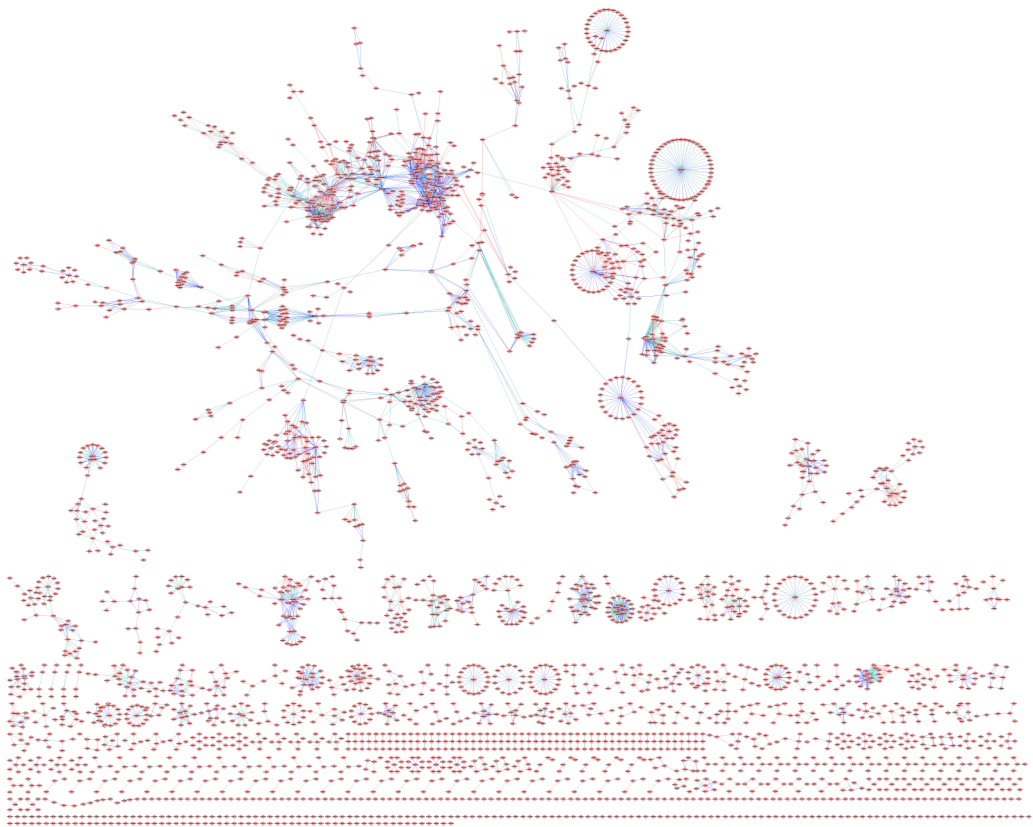

Supplement: Additional file 3 — MF-BP Association Network of human. MF-BP Association Network of human. The nodes represent terms and the edges represent the associations discovered by CroGO. The human MF-BP association network has a large subgraph occupying 50% of total edges. [file 1471-2105-14-S2-S15-S3.pdf]

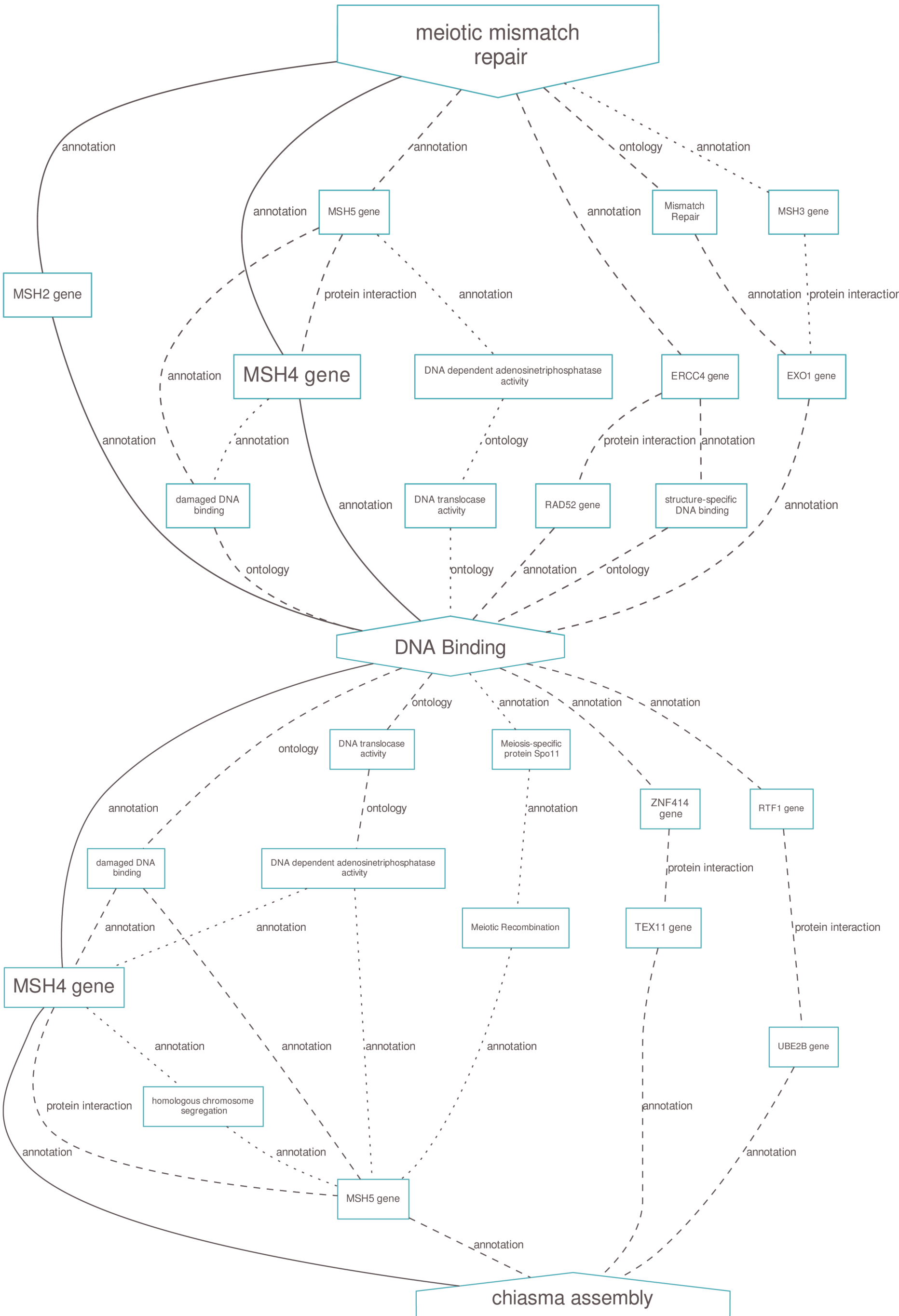

Supplement: Additional file 5 — genes and concepts that connect "DNA binding", "meiotic mismatch repair" and "chiasma assembly". The genes and concepts that connect "DNA binding", "meiotic mismatch repair" and "chiasma assembly". The figure was generated with BioGraph [40]. [file 1471-2105-14-S2-S15-S5.pdf]
